# Advances in the Development of Anti-*Haemonchus contortus* Vaccines: Challenges, Opportunities, and Perspectives

**DOI:** 10.3390/vaccines8030555

**Published:** 2020-09-22

**Authors:** Muhammad Ehsan, Rui-Si Hu, Qin-Li Liang, Jun-Ling Hou, Xiaokai Song, Ruofeng Yan, Xing-Quan Zhu, Xiangrui Li

**Affiliations:** 1 State Key Laboratory of Veterinary Etiological Biology, Key Laboratory of Veterinary Parasitology of Gansu Province, Lanzhou Veterinary Research Institute, Chinese Academy of Agricultural Sciences, Lanzhou 730046, China; mehsan124@gmail.com (M.E.); grishu0707@gmail.com (R.-S.H.); liangqinli701@163.com (Q.-L.L.); houjunling@caas.cn (J.-L.H.); 2 MOE Joint International Research Laboratory of Animal Health and Food Safety, College of Veterinary Medicine, Nanjing Agricultural University, Nanjing 210095, China; songxiaokai@njau.edu.cn (X.S.); yanruofeng@njau.edu.cn (R.Y.); lixiangrui@njau.edu.cn (X.L.); 3 College of Veterinary Medicine, Shanxi Agricultural University, Taigu 030801, China

**Keywords:** *Haemonchus contortus*, immune responses, host–parasite interactions, vaccine

## Abstract

The gastrointestinal nematode parasite *Haemonchus contortus* (*H. contortus*) is a resident of tropical and subtropical regions worldwide that imposes significant production losses, economic losses, and animal health issues in the small ruminant industry, particularly sheep and goats. Considerable efforts have been made to understand how immunity is elicited against *H. contortus* infection. Various potential vaccine antigens have been tested by different methods and strategies applied in animal models, and significant progress has been made in the development of vaccines against *H. contortus*. This review highlighted and shared the knowledge about the current understanding of host immune responses to *H. contortus* and ongoing challenges in the development of a protective, effective, and long-lasting vaccine against *H. contortus* infection. We have also pinpointed some achievements and failures in the development and testing of vaccines, which will establish a road map for future research directions to explore new effective vaccine candidates for controlling and preventing *H. contortus* infection.

## 1. Introduction

Haemonchosis/barber’s pole disease is a socio-economically important disease caused by one of the most pathogenic nematode parasites of ruminants (cattle, sheep, and goat) in tropical and subtropical areas of the world [1]. *Haemonchus contortus* (*H. contortus*) infection is clinically characterized by chronic wasting and anasarca, severe anemia and hypoproteinaemia (loss of 200–600 mL blood/day), decreased wool production, poor carcass quality, submandibular edema (bottle jaw), and even death of infected animals. Small ruminants, particularly sheep and goats, are important livestock species, contributing greatly to the agricultural economy of the world by producing meat, milk, skins, and wool [2,3,4,5]. It was estimated that the annual economic losses caused by *H. contortus* to the livestock industry accounted for $30–300 million globally [2,3]. The primary control practices against parasitic nematode *H. contortus* are still based on the application of anthelmintics; however, the excessive and uncontrolled use of a number of anti-parasitic drugs over many decades has led to the widespread emergence of drug resistance by the parasite [6]. At the end of the 20th century, anthelmintic resistance to all classes of broad-spectrum anthelmintics such as benzimidazoles, levamisole, pyrantel, morantel, avermectins/milbemycins, closantel, imidothiazoles-tetrahydropyrines, and macrocyclic lactones were reported in different parts of the world [7,8,9]. The emergence of drug resistance stimulates the demand for novel anti-parasitic vaccines and the development of powerful immunological approaches to control helminths infection. In this context, a deep insight into the biology of the *H. contortus* parasite through knowledge of immune responses during parasite–host interactions and cost-effective means of controlling haemonchosis is urgently needed [10].

**Figure 1:**
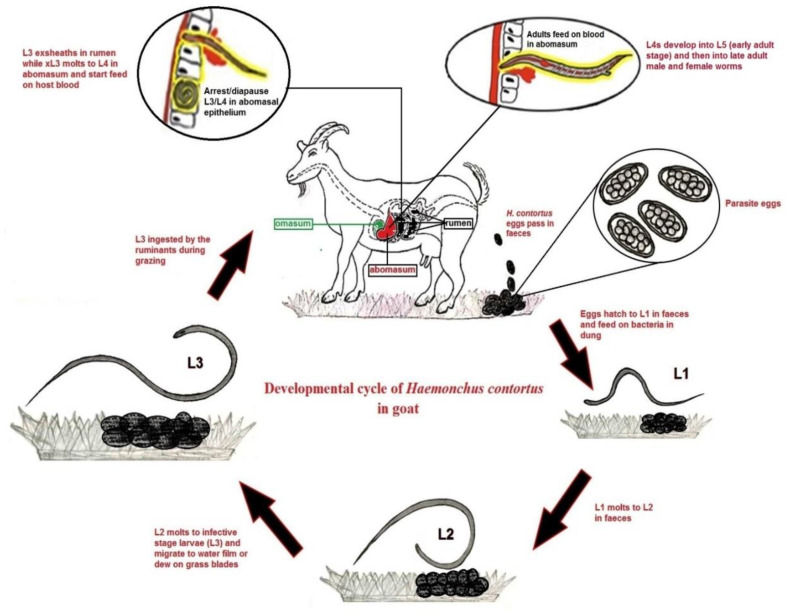
Developmental life cycle of *Haemonchus contortus* parasite in goat.

The life cycle of *H. contortus* starts when adult male and female worms reproduce sexually in the host abomasum with an impressive output per female worm estimated from 5000 to 15,000 eggs/day. The eggs embryonate into first-stage larvae (L1) and second-juvenile stage larvae (L2) within four to six days, and start feeding on bacteria in the dung. Under optimal conditions of 24–29 °C, the L2 larvae moult into the third stage (L3), but they retain the cuticle from the previous moult. During grazing, the infective stage L3 larvae reach the abomasum, shed their cuticle, and burrow into the internal layer of the abomasum and ex-sheath into L4 (pre-adult) stage, usually within 48 h, and finally develop to early (L5) and then late adult worms, which start feeding on blood (Figure 1). During the infective L3 or early L4 larval stages, parasites do not proceed directly to the next stage; instead, they remain in the gastric glands of the abomasum. This phenomenon is called developmental arrest (hypobiosis/diapause), and it is described as a “developmental stage in which the parasite becomes dormant, does not cause disease, and metabolically remains inactive” [11]. If the conditions outside the host are unfavorable for parasite development, the portion of hypobiotic worms is usually higher so that any egg shed into the environment would be unlikely to develop and survive [12]. This common mechanism of many parasitic worms, including *H. contortus*, depends on external factors and the immune responses of hosts as well, and also the genes involved in this phenomenon might serve as key antigens for new drug/vaccine targets in the control and prevention of *H. contortus* infection.

## 2. Importance of Anti-*H. contortus* Vaccine

Significant improvements have been made during the past two decades for identifying several antigens from *H. contortus*, and their protective efficiencies were evaluated as recombinant subunit/and DNA vaccines [13,14,15]. The potential efficacies of *H. contortus* gut proteases as vaccine components were investigated previously, including cysteine protease with fibrinogenolytic properties [16], an aminopetidase H11 [17], and gut membrane glycoprotein complex (H-gal-GP) [18], which is a gut protein complex containing metalloendopeptidase [19] and aspartyl protease activities [20]. Among these, a 110 kDa integral membrane glycoprotein H11 was found as a potential candidate antigen with >75% reduction in abomasal worm burden (AWB) and >90% reduction in fecal egg counts (FECs). As per our knowledge, a vaccine (Barbervax^®^, WormVax) containing two native integral gut membrane proteins from *H. contortus* (H11 and H-gal-GP) was recently licensed for commercial use in Australia [10]. Previously, an effort was made to evaluate the possible safety risks of Barbervax^®^ vaccine, and its serologic outcomes with significant antibody titers in vaccinated animals were determined [21]. The efficacy of the Barbervax^®^ vaccine was evaluated in the periparturient ewes under two different nutritional supplementations, which showed that vaccinated ewes conferred an 80% reduction in FECs and higher antibody titers, and it also suggested that sustainable control against *H. contortus* infection required a combined protective effect of vaccination along with improved nutrition [22]. This vaccine combination draws some disadvantages over large-scale application because of the repeated vaccination requirement to stimulate high antigen-specific and long-lasting circulating antibodies levels, which limited its access to the global market. In recent progress, immunological control of the *H. contortus* parasite via vaccination with soluble or recombinant proteins underpinned new insights into the biology of the parasite–host interactions [15]. Vaccines are a promising control strategy against parasites; however, the extensive genetic variation and immunoregulatory characteristics of parasites obviously hinder vaccine development [23]. Therefore, for the discovery of an effective, safe, and durable vaccine against *H. contortus*, researchers have been focusing on the development of molecular-based vaccine targets that are efficient against *H. contortus* infection and the utilization of advanced molecular approaches for structural and functional studies on vaccine candidates.

## 3. Mechanisms of Helminths-Associated Immunity

The immune responses to helminth parasites within their host are quite complex and characterized into two different stages: the immune responses to infective larvae and the immune responses against adult parasites. The T-helper cell type 2 (Th2) responses are considered the most effective resistance against helminths associated with high levels of serum and mucosal immunoglobulin-A (IgA), E (IgE), G (IgG), and eosinophilia at the site of infection [24,25,26]. The resistance against *H. contortus* infection is mainly associated with the breed and age of the host, as well as previous exposure to parasitic infection; especially, lambs are more susceptible to parasitism during the first 6 months of their lives, as they cannot develop immunity against *H. contortus*. This could differentially happen in older sheep, which usually can handle a moderate infection due to acquired immunity; however, this immunological phenomenon still not well understood. Several events are required for the clearance of nematode from the immunized host, including the activation of nonspecific defense mechanisms and the recognition of somatic and excretory/secretory (ES) antigens by dendritic cells (DCs), which act as antigen-presenting cells (APCs) for T cells [27]. The effector constituents of immunity are ‘‘allergic” inflammatory responses involving mucosal mast cells and eosinophils that secrete inflammatory mediators, which suppress egg production, prevent infective larvae and adult worm establishment, paralyze worm motility, and possibly mystify incoming larvae [28,29].

**Figure 2:**
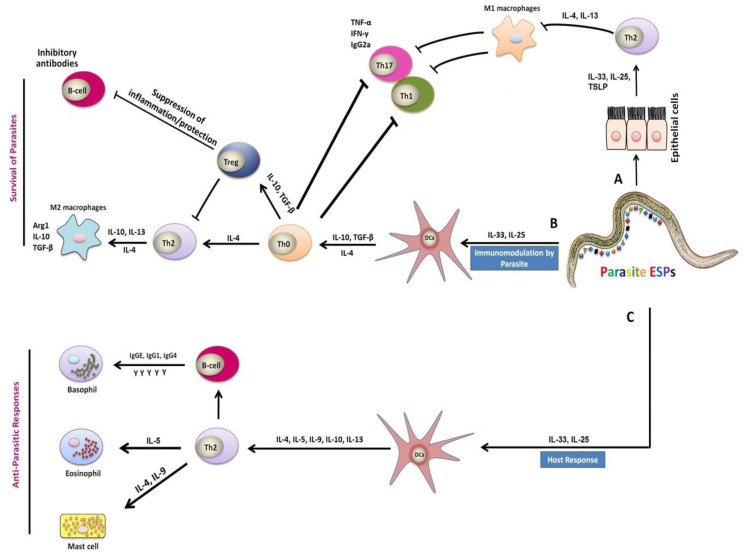
Mechanism of immune responses during host–parasite interactions. (**A**) Parasitic infection cause injuries to epithelial cells, which in turn stimulated and activated various aspects of the type 2 immune response with the generation of IL-25, IL-33 cytokines, and the Thymic Stromal Lymphopoietin (TSLP) cells. Macrophages exposure to the anti-inflammatory cytokines interleukin (IL)-4 or IL-13 lead to suppression of the classical activation of macrophages (M1) and stimulated expansion of the M2 phenotype [42]. (**B**) The excretory/secretory products (ESPs) released by parasite, induced immune responses linked with production of IL-25 and IL-33 cytokines, which are capable of inhibiting the activation and maturation of dendritic cells (DCs), and inducing the expansion of functional regulatory T cells (Tregs) [41]. Differentiation of alternatively activated macrophages (AAMs) induced by IL-4 and IL-13 can inhibit the proliferation of Th1, Th17, and Th2 cells. Thus, these cells have strong anti-inflammatory properties, manifested by the secretion of immunoregulatory cytokines IL-10 and TGF-β, as well as other related genes [40]. (**C**) The parasite-induced Th2 immune responses together with release of IL-25 and IL-33 cytokines are also responsible for the activation of DCs after the recognition of antigens by receptors on the DCs of the host [43]. These responses included IL-4, IL-5, IL-9, IL-10, and IL-13 secretions in addition to the production of immunoglobulin G (IgG) and immunoglobulin E (IgE) by B cells, and the activation of effector cells such as basophils, eosinophils, and mast cells, which caused secretions of inflammatory mediators responsible for parasite expulsion [31,32,33,34].

Generally, in case of helminths infection, Th2 immune responses are the part of the humoral responses associated with the specific expression of cytokines at the site of infection and cytokines acting as critical mediators for any immunological response, which evolved in CD4+ T cells stimulation and differentiation [27,30]. Helminths infection elicited strong Th2 cell responses associated with significant productions of interleukin (IL)-4, IL-5, IL-9, IL-10, IL-13, IL-25, and IL-31 [31,32,33] (Figure 2). Furthermore, these responses were also associated with high levels of IgE, IgG1, IgG4, and stable eosinophil and mast cell responses [34] (Figure 2). The cytokines IL-4 and IL-13 were considered as key regulators in humoral immunity, inducing B-cell class switching to IgE and regulating major histocompatibility complex (MHC) class II production [35]. These cytokines were involved in increased smooth-muscle-cell contractility, stimulation of epithelial cell permeability, and enhanced mucous production by goblet cells [35]. Mast cells, eosinophils, specific antibodies, and inhibitory molecules were generally involved in immunity to *H. contortus* parasite [26,36] (Figure 2). Some previous studies have shown that Th2 immune responses associated with helminths infection were characterized by the induction of IL-4, IL-13, IgG, IgG1, and IgE antibodies in rats [37,38], and the generation of IL-3, IL-4, IL-5, and IL-13 in mice [39]. Some studies reported that helminths have established various strategies to escape or modulate the host immune responses with advantages on both sides; for example, a modification in the Th2 response toward immunosuppression takes place as a result of the upregulation of regulatory T cells (Tregs), which suppressed protective Th2 as well as inflammatory Th1 responses in *Ancylostoma caninum* infection [40]. Furthermore, helminth-mediated Th2 responses could also prevent the harmful inflammatory Th1 responses by inducing suppressive Tregs, which contributed to the formation of IL-10 and transforming growth factor-β (TGF-β) cytokines. An earlier investigation has shown that *Trichinella spiralis* excretory/secretory products (ESPs) suppressed in vitro DCs maturation and induced the inflation of functional Tregs induced by both S- and R-form lipopolysaccharide (LPS) [41].

## 4. Genomic and Proteomic Profile Exploited from *H. contortus*

During the last two decades, deep insights into the molecular biology of the most significant parasitic nematode *H. contortus* have explored a large number of novel vaccine components by utilizing genomic and proteomic tools. Genome-wide transcriptomic data from all the key developmental stages of *H. contortus* revealed 23,610 protein-coding genes involved in host–parasite interactions, development, reproduction, immunity, and disease [44]. A comprehensive proteomic analysis using matrix-assisted laser desorption ionization time-of-flight mass spectrometry (MALDI-TOFMS or MALDI-TOF-MS/MS) and liquid chromatography tandem mass spectrometry (LC-MS/MS) pointed out 107 identities from 102 spots [45], including novel groups of proteins, such as aminopeptidases (H11), zinc metalloproteases, serine and aspartic proteases [46,47,48], and the most important immunogenic vaccines components of *H. contortus* ESPs (HcESPs) such as Hc-15, Hc-24, Hc-40, and apical gut proteins (GA1) [45,49,50]. Furthermore, in LC-MS/MS analysis, ≈4400 unique proteins were identified at initial host and parasite interaction (3 days post infection) [51]. Differentially expressed proteins between L3 and xL3 stages of *H. contortus* analyzed by two-dimensional differential gel electrophoresis (2D-DIGE) recognized 2200 proteins identities, and 124 of them exhibited between L3 and xL3 [52]. In a previous in vivo analysis of HcESPs, a total of 407 interacting proteins were identified ranging from 13 to 180 kDa; out of them, 47 proteins were shared in all developmental stages (L3 to adult) [53]. Whole protein extracts from male and female *H. contortus* identified 23 immunogenic, 132 female-specific, and 129 male-specific proteins of adult *H. contortus* parasite [54]. Furthermore, a proteomic study revealed a substantial transition in proteins profile from the free living to adult phase of the *H. contortus* parasite, in which a total of 2487 differential proteins were analyzed from eggs, L3s, L4s, and adult males and females [55]. The most recent identification of the secretome from L3s, L4s, and adult male and female developmental phases (stages/sexes) was carried out by LC-MS/MS analysis, in which 878 novel ES proteins were identified mainly involved in acquisition, nutrient digestion, and host–parasite interactions [56]. Herein, the purpose is to underpin the research progress on the identification of genomic and proteomic profiles exploited from *H. contortus*, which will provide a major resource to the scientific community for a wide range of epidemiological, genomic, and biological investigations and aid in the development of new drugs/vaccine interventions against *H. contortus*.

## 5. Historical Account of *H. contortus* Vaccines

Over the past few decades, significant efforts have been made to develop a vaccine against *H. contortus*. A large number of vaccine candidates were tested and have been under consideration by using various vaccination strategies, including recombinant subunit vaccines, DNA vaccines, and protein vaccines to govern their efficacy for protective immune responses against *H. contortus* infection. The commercial development of vaccines against helminths started as early as the 1950s, which resulted in the development of commercially available vaccines for a parasitic nematode named “Dictol”. However, later on, this was not effective against *H. contortus* infective larvae under field conditions. Therefore, the researches shifted toward the identification of immunogens from ESPs, as well as on gut-derived “hidden” antigens for the development of vaccines to combat haemonchosis. The vaccine trials for *H. contortus* using both ES and hidden antigens have been successfully employed as native vaccines, which gave a significant level of protection against haemonchosis with 70–95% reductions in FECs [13,57].

### 5.1. Vaccinations with Gut-Derived/Hidden Antigens

The glycoprotein complex derived from the gut of *H. contortus* (H-gal-GP) was found to be a highly protective immunogenic and vaccine candidate, which conferred >90% reduction in FECs and ≈70% reduction in worm output in a vaccination trial [58]. The H-gal-GP is a complex of the zinc metalloproteinases (MEPs) family of proteins, pepsinogen-like aspartyl proteinases, family of galectin proteins, as well as thrombospondin-like and cystatin components. The protective capacity of H-gal-GP was considered due to its glycan fractions rather than other structures in the complex [13]. Further studies on proteins belonging to the galectin family have also validated that galectins from *H. contortus* male and female worms (Hco-gal-m/f) potentially reduced FECs and AWB by 48% and 46%, respectively. The results highlighted that galectins could be protective vaccine antigens against *H. contortus* infection [59]. Other than H-gal-GP, an integral membrane glycoprotein complex designated as H11, belonging to a family of aminopeptidases derived from gut structure of blood-feeding nematode parasite *H. contortus*, has been well demonstrated in terms of its five isoforms named H11-1 to H11-5 [60]. Among the H11 complex, H11-1 is a highly protective immunogen abundantly found in the infective L3s stage and in various immunization trails; it produced a mean protective efficiency with 72% and 82% reductions in AWB (female and male worms), respectively, and a 91% reduction in FECs [10]. Furthermore, a combination of *Haemonchus* gut-derived protein-enriched fractions of H-gal-GP or H11 produced significant levels of protection from productive losses as well as parasite transmission, indicating that an antigen-specific serum IgG level was associated with vaccine efficacy [10,61]. This vaccine combination draws some disadvantages over large-scale application because of the repeated vaccination requirements to stimulate high antigen-specific and long-lasting circulating antibodies levels. Therefore, the development of an effective, durable, and safe vaccine relying on processing and production technology innovations is urgently needed.

### 5.2. DNA-Based Vaccination

This technology is used to deliver a genetically engineered DNA of a specific antigen so that immune cells can be directly exposed to the antigen and produce a wide range of protective immune responses. DNA vaccines have potential advantages over conventional vaccines, including the ability to induce a wider range of immune responses. Vaccines are the most effective means of disease control in human as well as in productive animals, whereas in the case of animal parasitic diseases, the development of vaccine is still beginning. The vaccine development against *H. contortus* requires high levels of antigen-specific antibodies for successful protection. Previously, a number of DNA vaccination trials showed that immunogenic fragments of H11, H11-1, and caprine IL-2 served as protective vaccine antigens, conferring a 47% reduction in AWB and 57% reduction in FECs upon the vaccination of eight to ten-month-old goats [62]. Another study was also done on animals of similar age, in which goats immunized with gene-encoding HC29 together with the glutathione peroxidase (GPX) conferred 36% reductions in FECs and AWB along with high levels of HC29-specific antibodies (IgG and IgA) and escalations of the CD4+ T lymphocytes population [63]. Further DNA vaccine studies were also assessed using two different antigens from *H. contortus* such as glyceraldehyde-3-phosphate dehydrogenase (GAPDH) and Dim-1 coupled with pVAX1 recombinant plasmids and administered in 10-month-old goats. The vaccination trial induced 35% and 38% reduction in FECs and AWB, respectively with GAPDH in addition to increases in antigen-specific serum IgG and IgA levels and the CD4+ T lymphocyte population. In contrast, vaccination with Dim-1 induced a significant level of protection with a 46% and 51% decrease in FECs and AWB, respectively [64,65].

### 5.3. Protein-Based Vaccination

During the last two decades, considerable contributions have been made to identify several potential vaccine antigens from *H. contortus* associated with the stimulation of prominent levels of host protective immunity [13,14]. During blood feeding stages, *H. contortus* releases a number of molecules/antigens into the host environment designated as ESPs [49,66,67]. The existence of parasites within the host territory required producing a range of molecules that interfere with the host’s defense system [68]. The ability of helminths to modulate the immune system strengthens their longevity within the host [32]. The body of nematode parasites is mainly associated with two categories of molecules: the first type is the ES products (soluble), and the second type is comprised of those antigens attached at external surfaces or within the parasite (so-called somatic antigens). The ES antigens that are exposed to somatic antigens but induced an immune response in the host during the course of infection are designated as natural antigens, while antigens that do not induce an immune response are called hidden antigens [69]. For an antigen to be a good vaccine target, parasite target antigens have to be exposable to antibodies and probably other immune response components. Some ES molecules are associated with the gut surface of parasites and released into host blood during the blood-feeding behavior of some parasites. HcESPs contain a source of potential vaccine targets because of their ability to induce up to 90% protection in sheep from *H. contortus* infection [45].

These molecules have been shown to stimulate a considerable level of protection against haemonchosis. Previously, two adult somatic extracts enriched in ES-15kDa and ES-24kDa were evaluated for their protective efficiency against challenge infection and yielded promising outcomes with reductions in FECs and AWB by 99.9% and 97.6%, respectively [70], whereas pure preparations of these antigens (ES-15 and ES-24 proteins) claimed protection efficiency of 77% and 85% for fecal egg output and worm numbers, respectively [49]. In addition, some studies based on *H. contortus* gut membrane antigen as a molecular vaccine showed a significant reduction in FECs by >90% and AWB by 72–80% in vivo [71,72]. Recently, Bu and colleagues have proven that another vaccine target antigen (rHcftt-2) identified previously as a vital-interacting protein to host peripheral blood mononuclear cells (PBMCs) reduced FECs and AWB by 26.46% and 32.33%, respectively [73]. Therefore, these studies have provided directions for the search of potential ES molecules with the best protective efficiency for the development of an effective vaccine in the near future.

## 6. Binding Proteins as Vaccine Antigens and Their Immunological Aspects

During the host–parasite relationship, parasites excrete and secrete large numbers of molecules into the host, which perform adversative immune-regulatory functions upon binding to host immune cells [74]. The ES products contain many proteins as immunogens; for example, 55 kDa glycoprotein showed immunomodulatory functions by inhibition of the host neutrophils [75]. Moreover, a purified 66 kDa adult *H. contortus* ES antigen inhibited monocyte function by the decreased production of hydrogen peroxide and nitric oxide in vitro [66]. Previously, the chemotactic activity of eosinophils and neutrophils in response to HcESPs was also reported [76]. Some in vitro studies reported that parasitic ESPs had direct effects on the cultured cells or tissues, such as the inhibition of acid secretion [77]. Additionally, the recombinant *H. contortus* galectin from male (rHco-gal-m) was recognized in the serum of naturally infected goat with *H. contortus* and reported that the binding of this protein to T cells and monocytes exhibited immunomodulatory capability, such as the expression of MHC II molecules, a decrease in T cell activation and proliferation, and the augmentation of T cells apoptosis, including several signaling pathways [78].

It is crucial to understand the mechanisms of the immune regulation during parasite invasion by investigating the interaction between parasite molecules and host cells. In this regard, the host PBMCs are considered as effectors during immune regulation, as they are mixtures of subpopulations, including lymphocytes (T cells, B cells, and natural killer (NK) cells), monocytes and DCs [78]. Moreover, the effects of recombinant *H. contortus* galectin peptides from both male and female (rHco-gal-m/f) on the apoptosis of goat PBMCs were investigated [79]. Furthermore, a joint study of proteomic and transcription analysis demonstrated that rHco-gal-m/f could bind on the surface of goat PBMCs, which promotes the suppressive inflammatory response to facilitate the immune evasion of *H. contortus* [80]. In the recent investigation, the interaction of transmembrane protein 147 (TMEM147) with galectins mediated cell proliferation, cell apoptosis, and cytokine transcription in goat PBMCs [81]. Similarly, it was noted that TMEM147 together with transmembrane protein 63A (TMEM63A) was also involved in the regulation of galectin on phagocytosis and nitric oxide production by goat PBMCs [82]. For better understanding the diverse range of biological activities of galectins from parasitic nematodes, TMEM63A was declared as a binding receptor for N-terminal CRD (MNh) and TMEM147 for C-terminal CRD (MCh), which enable Hco-gal-m to bind on the surface of goat PBMCs more efficiently [83].

In this review, we have also summarized the immunoregulatory dynamics of different HcESPs after binding to host immune cells (Table 1). Research showed that the combined effects of HcESPs displayed suppressive potential on the goat PBMCs in vitro by inhibiting the IL-4 and interferon gamma (IFN-γ) productions, increased the suppressive cytokine IL-10, enhanced the inflammatory modulator IL-17, suppressed the production of chemical factor nitric oxide, decreased the cell proliferation, and activated the cell migration [84]. Meanwhile, the interaction of *H. contortus* ES proteins secreted at different developmental stages of parasites was also reported in vivo using liquid chromatography-tandem mass spectrometry [53]. This study identified the secreted *H. contortus* 14-3-3 isoform 2 protein as an interacting protein to goat PBMCs in all parasitic stages, and in later investigation, it was found that an immunoregulatory recombinant protein of *H. contortus* (rHcftt-2) decreased the production of IL-4 and suppressed the proliferation of goat PBMCs in vitro [85]. In addition, two low molecular weight ES proteins from *H. contortus* (rHcES-24 and rHcES-15) were also evaluated for their immune regulatory potential, and it was found that immune interactions of both HcES-24 and HcES-15 with goat PBMCs significantly altered the host cells’ immune responses [86,87]. The binding of recombinant *H. contortus* arginine kinase (rHc-AK) to host cells decreased the cell proliferation and increased the apoptosis of host immune cells at considerable manners [88]. The serine/threonine–protein phosphatases (STPs), as integral constituents of parasitic ES proteins, were involved in the upregulation of various interleukins associated with protective immune responses, and the number of immune functions was altered, too [89]. Another antigen from HcESPs (rMiro-1) had stimulatory activities on goat PBMCs by promoting cell proliferation, along with the augmentation of IL-2, IL-4, and IL-17 cytokines [90]. A previous research highlighted that an interacting protein of HcESPs (rHcARF1) modulated PBMCs functions by the upregulation of IL-4, IL-10, and IL-17 cytokines in vitro [91]. It was indicated that elongation factor 1 alpha (rHcEF-1α) played essential roles in the functional regulations of HcESPs on goat PBMCs by modulating IL-4, IL-17, IFN-γ, and TGF-β1 cytokine levels, in addition to some suppressive activities on host immune cells [92]. Apart from the discrete functional potency of different constituents of HcESPs on goat PBMCs, a recent research on tropomyocin protein showed the suppressive potential of rHc-TpMy on goat PBMCs by suppressing various immune functions of host PBMCs [93]. Recently, we have explored a novel immune modulator from HcESPs, which improved the IFN-γ, IL-4, and IL-2 cytokines production of host immune cells [94]. In addition to that, ES proteins were found to have distinct immunomodulatory and suppressive regulatory properties during host–parasite interactions, which suggested that the discoveries of these novel antigens might be helpful in anti-parasitic vaccine development in the near future.

## 7. Challenges to the Development of Effective Vaccines

A number of vaccine trials have been undertaken for more than three decades in the search of effective vaccine development against *H. contortus*, and regardless of employing multiple approaches to test many antigens, none of them has been proven to fully eliminate or stop transmission of the parasite. Vaccine against haemonchus requires minimizing the adult worms output early in the grazing season, as well as either by reducing the establishment of infective larvae or by killing established worms. The scarcity of anti-parasitic recombinant vaccines indicated that several impediments in the development of an effective vaccine still exist.

### 7.1. Diversity Within H. contortus Parasite

*H. contortus* parasite has been potentially studied in terms of genetic diversity. Many research fields including epidemiology, anthelmintic resistance, molecular diagnostics, drugs/vaccines development, and control require better understanding of its genetic diversity. An analysis of its population genetic structure revealed that the genetic diversity is particularly influenced by various factors, including the life history, population size, environmental or geographical barriers, and gene flow [97]. Thus, extreme genetic variations in *H. contortus* isolates within-population were observed, suggesting that it could be a result of large population sizes or high mutation rates [98]. It was also noted that extensive genetic diversity exists within and among *H. contortus* laboratory strains in population structures from different countries [99]. Some studies suggested that genetically different isolates have also been detected in sheep and goats from the same geographical region. Furthermore, five laboratory isolates of *H. contortus* from geographical regions of Australia were used to infect 10 sheep, and it was found that significant variations in the establishment rate increased in FECs and AWB [100]. Genetic diversity studies of *H. contortus* have revealed that variation in the pathogenicity occurs between different isolates of *H. contortus* collected from various areas of the United States [98]. The experimental infections with 18 different *H. contortus* isolates reported that three isolates had significantly reduced pathogenicity, whereas two isolates had increased pathogenicity to that of the control one [101]. Moreover, research studies involving heterologous challenge suggested that antigens associated with protective immunity are not necessarily conserved among species or even among isolates of the same species [102]. In case of vaccine development, issues related to genetic diversity in parasites and stage-specific immune responses demands a vaccine based on native antigens from multiple stages of the *H. contortus* parasite, which could be an alternative approach; for example, a vaccine combination of L3 surface antigen and hidden gut antigen from L4 and adult stages of *H. contortus* could promote the pre-seasonal protection of young animals by inducing natural immunity with L3 surface antigen and also diminish the requirement of post-seasonal booster dose [10]. This no doubt a challenging work, necessitating the collection of multiple parasitic stages from naturally infected host and identifying that a large profile of antigens definitely need financial support as well [103]. However, a successful development of an effective vaccine will surely benefit the whole world.

### 7.2. Host-Related Genetic Diversity

The assessment of antigen-related immune responses during the host–parasite relationship is intensely associated with genetic diversity within host species. It has been demonstrated previously that genetics contribute immensely to innate or acquired resistance within a herd of animals carrying nematode infection [57]. Generally, it was predicted that almost 100% immunity with vaccination could be achieved in animals with existing strong natural immunity against infection compared with those who have been involved in a parasite–host relationship for a long duration [104]. The host genetic variability influence highly on parasites eggs per gram (EPG) of grazing animals, and further study demonstrated that an increased eggs output in feces as well as the majority of parasite transmission occurs as a result of a small portion of highly vulnerable animals within the population [105]. Furthermore, it was also suggested that host pathogenicity and parasitism can be reduced only by the vaccination of a small percentage of susceptible hosts within the herd [57]. Host populations with low genetic diversity were more susceptible to infection with well-adopted parasite species. A growing body of evidence predicts that ecological alteration, global warming, pollution, as well as limitation in the geographical range of species may reduce host genetic diversity [106]. On the other hand, a high level of parasite dominancy increased in the homogeneous host populations with the co-infection of various parasitic species [107], rather than the interplay of a single parasite and host pair. During parasite–host interplay, the intensity and timing of infection might regulate the overall pathogenicity in the host population [106]. In addition, historical contact of same mechanism of infection by parasites is considered as an important factor for resistance to the parasitic species; however, from a conservation perception, global climate change poses a potential impact on genetic diversity within species [108]. Meanwhile, a potential association between emerging novel infections and the low genetic diversity of a population also exists. Therefore, management efforts toward preserving host genetic diversity alongside the vaccination of the most susceptible animals within a herd may improve the resistance ability against emerging parasitic diseases.

### 7.3. Multistage Complexity of Parasite

Similar to other parasitic species, *H. contortus* has a multistage life cycle, and at each stage, the parasite expresses a differential antigenic profile during progression [53]. However, the immune mechanism involved in a specific stage (L3, L4, and L5) of *H. contortus* may be unable to protect from infection caused by other stages, which poses inimitable challenges for vaccine development. Stage-specific-based vaccines comprised of single or double antigens may provoke immune responses with cross-protection deficiency. The complexity of life stages combined with substantial genetic variations raises uncertainties that vaccines based on a single antigen could confer the required long-lasting protection. Therefore, multiple immunogenic antigens from different *H. contortus* life cycle stages pooled into a multistage-specific cocktail might play a beneficial role in the future commercial vaccine development for the prevention of haemonchosis. Another factor that hampers the effective progress of vaccine development is lack of in vitro culture methods for appropriate stages of the parasites, which require maintained passage and persistent infection within their host. Furthermore, the ability of various parasitic species to modulate host immune responses in order to delay or inhibit resistance against the parasites is also a considerable challenge in vaccine development.

### 7.4. Composition of Protective Vaccine

Generally, during the vaccine development process, a major deficiency requiring attention is the need for induced immune responses durability and perfection in vaccine immunogenicity. In this context, adjuvants have been considered as key components in vaccine formulation for several reasons: to produce high antibodies and rapid immune responses through B cell differentiation with enhanced functionality and magnitude, to reduce cost by improving effectiveness, to improve biodegradability, safety, and stability, and also to lower the quantity of antigens per dose and the number of doses needed [109,110]. Among the numbers of adjuvants employed today, the choice of antigen–adjuvant complex is chiefly based on the equilibrium state between high immunogenicity and the lesser side effects in the vaccinated host. Different adjuvants applied during the vaccine delivery system and their outputs were evaluated in different host models [111]. However, this vaccine delivery system used in vaccine development has some drawbacks, such as glucopyranosyl lipid adjuvant (GLA), a Toll-like receptor agonist, has triggered a balanced IgG1/IgG2 response and promoted cytotoxic T lymphocytes-based strong Th1 responses in immunized hosts [112]. Vaccines coupled with adjuvants such as Montanide™, saponin, and Freund’s Complete have been utilized in different vaccination trials; however, the administration of Montanide™ and saponin caused adverse effects in animal models such as tissue damage and inflammation at the site of injection, leading to improper host immune responses [109,113]. Recently, a breakthrough in the vaccine delivery system offered a new promising alternative technology to conventional adjuvants called the “microencapsulation” of parasite antigens, which could be proven as a novel, effective, and durable vaccine delivery system; it approached various delivery routes and could possibly play immune regulatory roles [114]. Considering adjuvants as safe and effective means of vaccine delivery system, some studies exposed that the vaccination of lambs with native or recombinant *H. contortus* 23 (Hc23, rHc23) antigen coupled with aluminum hydroxide as adjuvant elicited 70–80% reductions in FECs and AWB against challenged infection [115,116]. Another study also confirmed that the protective efficacy of rHc23 coupled with adjuvant (aluminum hydroxide) conferred an 80% and 70% reduction in FECs and AWB, respectively [117]. Furthermore, it was determined that native Hc23 reduced FECs and AWB by 70% and 67% respectively when co-administered with aluminum hydroxide, and it showed reductions of 85% and 87% when coupled with bacterial lipopolysaccharide, respectively [115].

## 8. Future Perspectives

Extensive progress has been made around the globe in the search of potential antigens from *H. contortus*, which are of vaccine significance, and various antigens were tested for vaccine efficacy in animal models as well. However, there is still a lack of an effective vaccine to prevent *H. contortus* infection or retard its transmission. Research studies on helminths parasites have explored most of the complicated strategies of parasitic existence and survival within hosts, such as adaptive changes in its stage-specific developmental biology, anthelmintics resistance, and escape from host immune responses, which all lie under the genetic diversity of the *H. contortus* parasite. The recent development of a more reliable, sensitive, and specific diagnostic tool that detected infection at a very early stage provided a ray of hope for the livestock industry to cope with challenge of *H. contortus* parasites. Omics approaches can potentially magnify our understandings on parasite epidemiology and biology, along with the identification of novel targets for serodiagnostics and control of the *H. contortus* parasite. Many efforts have been made in investigations on diapause-related genes and proteomics/and transcriptomics-based key molecules involved in mechanisms of immune regulation, and their protective efficiencies provided satisfactory knowledge about molecular mechanisms in developmental changes and immune invasion of the parasite during parasite–host interactions. However, technology-oriented investigations are the way forward for further identifications of a new class of key immunotherapeutic compounds, and evaluation of their immunoregulatory characteristics are urgently needed. Recently, a breakthrough in the vaccine delivery system offered a new promising alternative technology to conventional adjuvants called the “microencapsulation” of parasite antigens, which could play a beneficial role in future commercial *H. contortus* vaccine development [114]. Furthermore, functional genomic research based on molecular advances and genome sequencing together with the most advanced genome editing technology CRISPR-Cas should be extended to the nematode parasite *H. contortus* in the future. With the wealth of genomic and transcriptomics data available, and CRISPR-Cas genome editing tools may form part of an improved alternative strategy in anti-*H. contortus* vaccine development [118].

## 9. Concluding Remarks

Since the global emergence of anthelmintics resistance, substantial progress has been made, and still, work is on the way to identify and evaluate protective efficiencies of various vaccine candidate antigens. However, none have shown robust and passable protective efficacy yet. Prospective impediments and elucidations during the developmental phase of an effective haemonchosis vaccine have been discussed. There is still the need to fully understand the immunopathology of *H. contortus* during parasite–host interactions for the accurate identification of better immune correlates and vaccination candidates, and most importantly, the precise choice of delivery vehicle or adjuvant for vaccination. Therefore, a deep insight into the immunopathology of *H. contortus* in interaction with domestic hosts (sheep, goats) is still needed to establish a road map in the development of vaccines, which will have a pivotal role in the prevention and control of *H. contortus* infection. Finally, the incorporation of the most advanced innovational strategies such as genomics, transcriptomics, proteomics, and metabolomics will boost up the progress of a more effective, safe, and durable vaccine against *H. contortus* in the near future.

## Figures and Tables

**Table 1:** Immunoregulatory dynamics of different *Haemonchus contortus* excretory/secretory proteins (HcESPs) after binding to host immune cells.

Antigen Name	Database ID	Life Stage	Host Immune Cells	Immune Functional Roles ↑(Increase), ↓(Decrease)	Ref.
rHco-gal-m	AY253330	L4 to adult	Monocytes	↑IL-10, ↓TNF-α, ↓MHCII, ↓apoptosis	[78]
			T cells	↓IL-6, ↓IL-10, ↓TNF-α, ↓proliferation, ↑apoptosis	
rHco-gal-m/f	AY253330/AY253331	L4 to adult	PBMCs	↓IL-2, ↓IL-4, ↓IL-6, ↓IL-8, ↑IL-10, ↓IFN-γ, ↓TNF-α, ↑TGF-β1, ↓migration	[80]
HcESPs	Whole proteins	L4 to adult	PBMCs	↓IL-4, ↑IL-10, ↑IL-17, ↓IFN-γ, ↓proliferation, ↓NO, ↑migration	[84]
rHcES-15	AY821552.1	L4 to adult	PBMCs	↑IL-4, ↑IL-10, ↑IL-17, ↓TGF-β1, ↓IFN-γ, ↑NO, ↑apoptosis, ↓migration, ↓proliferation	[87]
rHcES-24	AY821551.1	L4 to adult	PBMCs	↑IL-4, ↑IL-10, ↑IL-17, ↓IFN-γ, ↑migration, ↓proliferation, ↓NO	[86]
rHcftt-2	CDJ94531	L4 to adult	PBMCs	↓IL-4, ↑IL-10, ↑IL-17, ↑IFN-γ, ↑migration, ↓NO, ↓proliferation	[85]
rHc-AK	JX422018.1	L4 to adult	PBMCs	↑IL-4, ↑IL-10, ↑IL-17, ↑IFN-γ, ↓TGF-β1, ↑NO,↑apoptosis,↓migration, ↓proliferation	[88]
rHcSTP-1	GQ280010.1	Adults	PBMCs	↑IL-2, ↓IL-10, ↑TGF-β1, ↑IFN-γ, ↑IL-17, ↑migration, ↑apoptosis, ↑NO, ↓proliferation	[89]
rHcARF1	HF964523.1	L4 to adult	PBMCs	↑IL-4, ↑IL-10, ↑IL-17,↓IFN-γ, ↑NO, ↑migration, ↓proliferation	[91]
rMiro-1	CDJ96345.1	Adults	PBMCs	↑IL-2, ↑IL-4, ↑IL-17, ↑migration, ↑NO, ↑proliferation	[90]
rHCcyst-3	CDJ92568.1	Eggs to adults	Monocytes	↑IL-10, ↑TGF-β1, ↓TNF-α, ↓IL-1β, ↓IL-12p40, ↑NO, ↓phagocytosis, ↓MHCII	[95]
rHcAPI	KY284864.1	Eggs to adults	PBMCs	↑IL-4, ↑IL-10, ↑IFN-γ	[96]
rHcEF-1α	HF960353.1	L4+L5	PBMCs	↑IL-4, ↑IL-17, ↑TGF-β1, ↑IFN-γ, ↓IL-10, ↑migration, ↑apoptosis, ↓NO, ↑proliferation	[92]
HcMT-12	CDJ87424.1	L4+L5	PBMCs	↑IL-2, ↑IL-4, ↓IL-10, ↑IFN-γ, ↓TGF-β1, ↑migration, ↑NO, ↓proliferation	[94]
rHc-TpMy	HF965396	L4+L5	PBMCs	↓IL-4, ↓IFN-γ, ↑IL-10, ↑IL-17, ↑TGF-β1, ↓NO, ↓migration, ↑apoptosis, ↓proliferation	[93]

## References

[1] O’Connor L.J., Walkden-Brown S.W., Kahn L.P. (2006). Ecology of the free-living stages of major trichostrongylid parasites of sheep. Vet. Parasitol..

[2] Roeber F., Jex A.R., Gasser R.B. (2013). Impact of gastrointestinal parasitic nematodes of sheep, and the role of advanced molecular tools for exploring epidemiology and drug resistance—An Australian perspective. Parasites Vectors.

[3] Emery D.L., Hunt P.W., Le Jambre L.F. (2016). *Haemonchus contortus*: The then and now, and where to from here?. Int. J. Parasitol..

[4] Peter J.W., Chandrawathani P. (2005). *Haemonchus contortus*: Parasite problem No. 1 from tropics—Polar Circle. Problems and prospects for control based on epidemiology. Trop. Biomed..

[5] Roos M.H. (2009). Drug Resistance in the sheep nematode parasite *Haemonchus contortus*, mechanisms and clinical perspectives. Antimicrobial Drug Resistance: Clinical and Epidemiological Aspects.

[6] Saddiqi H.A., Riaz N., Sarwar M., Iqbal Z., Muhammad G., Nisa M., Shahzad A. (2011). Small ruminant resistance against gastrointestinal nematodes: A case of *Haemonchus contortus*. Parasitol. Res..

[7] Kaplan R.M., Vidyashankar A.N. (2012). An inconvenient truth: Global worming and anthelmintic resistance. Vet. Parasitol..

[8] Kaplan R.M. (2004). Drug resistance in nematodes of veterinary importance: A status report. Trends Parasitol..

[9] Prichard R. (2009). Drug resistance in nematodes. Antimicrobial Drug Resistance: Mechanisms of Drug Resistance.

[10] Nisbet A.J., Meeusen E.N.T., González J., Piedrafita D. (2016). Immunity to *Haemonchus contortus* and vaccine development. Adv. Parasitol..

[11] Michel J. (1974). Arrested development of nematodes and some related phenomena. Adv. Parasitol..

[12] Waller P., Rudby-Martin L., Ljungström B., Rydzik A. (2004). The epidemiology of abomasal nematodes of sheep in Sweden, with particular reference to over-winter survival strategies. Vet. Parasitol..

[13] Knox D.P., Redmond D.L., Newlands G.F., Skuce P.J., Pettit D., Smith W.D. (2003). The nature and prospects for gut membrane proteins as vaccine candidates for *Haemonchus contortus* and other ruminant trichostrongyloids. Int. J. Parasitol..

[14] Tak I.R., Dar J.S., Dar S.A., Ganai B.A., Chishti M.Z., Ahmad F. (2015). A comparative analysis of various antigenic proteins found in *Haemonchus contortus*—A review. Mol. Biol..

[15] Wang C., Li F., Zhang Z., Yang X., Ahmad A.A., Li X., Du A., Hu M. (2017). Recent research progress in China on *Haemonchus contortus*. Front. Microbiol..

[16] Boisvenue R.J., Stiff M.I., Tonkinson L.V., Cox G.N., Hageman R. (1992). Fibrinogen-degrading proteins from *Haemonchus contortus* used to vaccinate sheep. Am. J. Vet. Res..

[17] Smith T.S., Graham M., Munn E.A., Newton S.E., Knox D.P., Coadwell W., McMichael-Phillips D., Smith H., Smith W., Oliver J.J. (1997). Cloning and characterization of a microsomal aminopeptidase from the intestine of the nematode *Haemonchus contortus*. Biochimica Biophysica Acta (BBA).

[18] Smith W.D., Smith S.K., Murray J.M. (1994). Protection studies with integral membrane fractions of *Haemonchus contortus*. Parasite Immunol..

[19] Redmond D.L., Knox D.P., Newlands G., Smith W.D. (1997). Molecular cloning and characterisation of a developmentally regulated putative metallopeptidase present in a host protective extract of *Haemonchus contortus*. Mol. Biochem. Parasitol..

[20] Longbottom D., Redmond D.L., Russell M., Liddell S., Smith W.D., Knox D.P. (1997). Molecular cloning and characterisation of a putative aspartate proteinase associated with a gut membrane protein complex from adult *Haemonchus contortus*. Mol. Biochem. Parasitol..

[21] Vanhoy G., Carman M., Habing G., Lakritz J., Hinds C.A., Niehaus A., Kaplan R.M., Marsh† A.E. (2018). Safety and serologic response to a *Haemonchus contortus* vaccine in alpacas. Vet. Parasitol..

[22] Bassetto C., Almeida F., Newlands G.F.J., Smith W., Castilhos A., Fernandes S., Siqueira E., Amarante A. (2018). Trials with the *Haemonchus* vaccine, Barbervax^®^, in ewes and lambs in a tropical environment: Nutrient supplementation improves protection in periparturient ewes. Vet. Parasitol..

[23] Hewitson J.P., Maizels R.M. (2014). Vaccination against helminth parasite infections. Expert Rev. Vaccines.

[24] Lacroux C., Nguyen T.H.C., Andréoletti O., Prévot F., Grisez C., Bergeaud J.-P., Gruner L., Brunel J.-C., Francois D., Dorchies P. (2006). *Haemonchus contortus* (Nematoda: *Trichostrongylidae*) infection in lambs elicits an unequivocal Th2 immune response. Vet. Res..

[25] Shakya K., Miller J., Lomax L., Burnett D. (2011). Evaluation of immune response to artificial infections of Haemonchus contortus in Gulf Coast Native compared with Suffolk lambs. Vet. Parasitol..

[26] Balic A., Cunningham C.P., Meeusen E.N.T. (2006). Eosinophil interactions with *Haemonchus contortus* larvae in the ovine gastrointestinal tract. Parasite Immunol..

[27] Meeusen E.N.T., Balic A., Bowles V.M. (2005). Cells, cytokines and other molecules associated with rejection of gastrointestinal nematode parasites. Vet. Immunol. Immunopathol..

[28] Jones W., Emery D., McClure S., Wagland B. (1994). Changes in inflammatory mediators and larval inhibitory activity in intestinal contents and mucus during primary and challenge infections of sheep with *Trichostrongylus colubriformis*. Int. J. Parasitol..

[29] Emery D. (1996). Vaccination against worm parasites of animals. Vet. Parasitol..

[30] Anthony R.M., Urban J.F., Alem F., Hamed H.A., Rozo C.T., Boucher J.-L., Van Rooijen N., Gause W.C. (2006). Memory TH2 cells induce alternatively activated macrophages to mediate protection against nematode parasites. Nat. Med..

[31] Jackson J.A., Friberg I.M., Little S., Bradley J.E. (2009). Review series on helminths, immune modulation and the hygiene hypothesis: Immunity against helminths and immunological phenomena in modern human populations: Coevolutionary legacies?. Immunology.

[32] Maizels R.M., Yazdanbakhsh M. (2003). Immune Regulation by helminth parasites: Cellular and molecular mechanisms. Nat. Rev. Immunol..

[33] Wang L.J., Cao Y., Shi H.N. (2008). Helminth infections and intestinal inflammation. World J. Gastroenterol..

[34] Ditgen D., Anandarajah E.M., Meissner K.A., Brattig N., Wrenger C., Liebau E. (2014). Harnessing the helminth secretome for therapeutic immunomodulators. Biomed Res. Int..

[35] Anthony R.M., Rutitzky L.I., Urban J.F., Stadecker M.J., Gause W.C. (2007). Protective immune mechanisms in helminth infection. Nat. Rev. Immunol..

[36] Bricarello P., Gennari S.M., Oliveira-Sequeira T., Vaz C., De Gonçalves I.G., Echevarria F. (2004). Worm burden and immunological responses in Corriedale and Crioula Lanada sheep following natural infection with *Haemonchus contortus*. Small Rumin. Res..

[37] Wilkes C.P., Bleay C., Paterson S., Viney M. (2007). The immune response during a *Strongyloides ratti* infection of rats. Parasite Immunol..

[38] Paterson S., Wilkes C., Bleay C., Viney M.E. (2008). Immunological responses elicited by different infection regimes with *Strongyloides ratti*. PLoS ONE.

[39] Eschbach M.-L., Klemm U., Kolbaum J., Blankenhaus B., Brattig N., Breloer M. (2010). *Strongyloides ratti* infection induces transient nematode-specific Th2 response and reciprocal suppression of IFN-γ production in mice. Parasite Immunol..

[40] Ferreira I., Smyth D.J., Gaze S., Aziz A., Giacomin P.R., Ruyssers N., Artis D., Laha T., Navarro S., Loukas A. (2013). Hookworm excretory/secretory products induce interleukin-4 (IL-4)+IL-10+CD4+T cell responses and suppress pathology in a mouse model of colitis. Infect. Immun..

[41] Aranzamendi C., Franssen F., Rutten V.P.M.G., Fransen F., Langelaar M.F.M., Van Der Ley P., Van Putten J.P.M., Pinelli E. (2012). *Trichinella spiralis*—Secreted products modulate DC functionality and expand regulatory T cells in vitro. Parasite Immunol..

[42] Abdoli A., Ardakani H.M. (2020). Helminth infections and immunosenescence: The friend of my enemy. Exp. Gerontol..

[43] Mendlovic F., Flisser A. (2010). Dendritic cells in the gut: Interaction with intestinal helminths. J. Biomed. Biotechnol..

[44] Schwarz E.M., Korhonen P.K., Campbell B.E., Young N.D., Jex A.R., Riaz N., Hall R.S., Mondal A., Howe A., Pell J. (2013). The genome and developmental transcriptome of the strongylid nematode *Haemonchus contortus*. Genome Boil..

[45] Yatsuda A., Krijgsveld J., Cornelissen A.W.C.A., Heck A.J.R., De Vries E. (2003). Comprehensive analysis of the secreted proteins of the parasite *Haemonchus contortus* reveals extensive sequence variation and differential immune recognition. J. Boil. Chem..

[46] Rhoads M., Fetterer R. (1995). Developmentally regulated secretion of cathepsin L-like cysteine proteases by *Haemonchus contortus*. J. Parasitol..

[47] Shompole S., Jasmer D.P. (2000). Cathepsin B-like cysteine proteases confer intestinal cysteine protease activity in *Haemonchus contortus*. J. Boil. Chem..

[48] Karanu F., Rurangirwa F., McGuire T., Jasmer D. (1993). *Haemonchus contortus*: Identification of proteases with diverse characteristics in adult worm excretory-secretory products. Exp. Parasitol..

[49] Schallig H.D., Van Leeuwen M.A., Cornelissen A.W. (1997). Protective immunity induced by vaccination with two *Haemonchus contortus* excretory secretory proteins in sheep. Parasite Immunol..

[50] Dicker A.J., Inglis N.F., Manson E.D.T., Subhadra S., Illangopathy M., Muthusamy R., Knox D.P. (2014). Proteomic analysis of *Mecistocirrus digitatus* and *Haemonchus contortus* intestinal protein extracts and subsequent efficacy testing in a vaccine trial. PLoS Neglected Trop. Dis..

[51] Nagaraj S.H., Harsha H., Reverter A., Colgrave M.L., Sharma R., Andronicos N., Hunt P., Menzies M., Lees M.S., Sekhar N.R. (2012). Proteomic analysis of the abomasal mucosal response following infection by the nematode, *Haemonchus contortus*, in genetically resistant and susceptible sheep. J. Proteom..

[52] Wang F., Xu L., Song X., Li X., Yan R. (2016). Identification of differentially expressed proteins between free-living and activated third-stage larvae of *Haemonchus contortus*. Vet. Parasitol..

[53] Gadahi J.A., Wang S., Bo G., Ehsan M., Yan R., Song X., Xu L., Li X. (2016). Proteomic analysis of the excretory and secretory proteins of *Haemonchus contortus* (HcESP) binding to goat PBMCs in vivo revealed stage-specific binding profiles. PLoS ONE.

[54] Yan F., Xu L., Liu L., Yan R., Song X., Li X. (2010). Immunoproteomic analysis of whole proteins from male and female adult *Haemonchus contortus*. Vet. J..

[55] Wang T., Ma G., Ang C.-S., Korhonen P.K., Xu R., Nie S., Koehler A.V., Simpson R.J., Greening D.W., Reid G.E. (2019). Somatic proteome of *Haemonchus contortus*. Int. J. Parasitol..

[56] Wang T., Ma G., Ang C.-S., Korhonen P.K., Koehler A.V., Young N.D., Nie S., Williamson N.A., Gasser R.B. (2019). High throughput LC-MS/MS-based proteomic analysis of excretory-secretory products from short-term in vitro culture of *Haemonchus contortus*. J. Proteom..

[57] Smith W., Zarlenga D. (2006). Developments and hurdles in generating vaccines for controlling helminth parasites of grazing ruminants. Vet. Parasitol..

[58] Smith S.K., Smith W.D. (1996). Immunisation of sheep with an integral membrane glycoprotein complex of *Haemonchus contortus* and with its major polypeptide components. Res. Vet. Sci..

[59] Yanming S., Ruofeng Y., Muleke C.I., Guangwei Z., Lixin X., Li X. (2007). Vaccination of goats with recombinant galectin antigen induces partial protection against *Haemonchus contortus* infection. Parasite Immunol..

[60] Roberts B., Antonopoulos A., Haslam S.M., Dicker A.J., McNeilly T.N., Johnston S.L., Dell A., Knox D., Britton C. (2013). Novel expression of *Haemonchus contortus* vaccine candidate aminopeptidase H11 using the free-living nematode *Caenorhabditis elegans*. Vet. Res..

[61] Smith S., Pettit D., Newlands G., Redmond D., Skuce P., Knox D., Smith W. (1999). Further immunization and biochemical studies with a protective antigen complex from the microvillar membrane of the intestine of *Haemonchus contortus*. Parasite Immunol..

[62] Zhao G., Yan R., Muleke C.I., Sun Y., Xu L., Li X. (2012). Vaccination of goats with DNA vaccines encoding H11 and IL-2 induces partial protection against *Haemonchus contortus* infection. Vet. J..

[63] Sun W., Song X., Yan R., Xu L., Li X. (2011). Vaccination of goats with a glutathione peroxidase DNA vaccine induced partial protection against *Haemonchus contortus* infection. Vet. Parasitol..

[64] Han K., Xu L., Yan R., Song X., Li X. (2012). Vaccination of goats with glyceraldehyde-3-phosphate dehydrogenase DNA vaccine induced partial protection against *Haemonchus contortus*. Vet. Immunol. Immunopathol..

[65] Yan R., Sun W., Song X., Xu L., Li X. (2013). Vaccination of goats with DNA vaccine encoding Dim-1 induced partial protection against *Haemonchus contortus*: A preliminary experimental study. Res. Vet. Sci..

[66] Rathore D., Suchitra S., Saini M., Singh B., Joshi P. (2006). Identification of a 66 kDa *Haemonchus contortus* excretory/secretory antigen that inhibits host monocytes. Vet. Parasitol..

[67] Marcilla A., Trelis M., Cortés A., Sotillo J., Cantalapiedra F., Minguez M.T., Valero M.L., Del Pino M.M.S., Muñoz-Antoli C., Toledo R. (2012). Extracellular vesicles from parasitic helminths contain specific excretory/secretory proteins and are internalized in intestinal host cells. PLoS ONE.

[68] Nagaraj S.H., Gasser R.B., Ranganathan S. (2008). Needles in the EST haystack: Large-scale identification and analysis of Excretory-Secretory (ES) proteins in parasitic nematodes using Expressed Sequence Tags (ESTs). PLoS Neglected Trop. Dis..

[69] Munn E.A. (1997). Rational design of nematode vaccines: Hidden antigens. Int. J. Parasitol..

[70] Schallig H.D.F.H., Van Leeuwen M.A.W. (1997). Protective immunity to the blood-feeding nematode *Haemonchus contortus* induced by vaccination with parasite low molecular weight antigens. Parasitology.

[71] Jasmer D.P., McGuire T.C. (1991). Protective immunity to a blood-feeding nematode (*Haemonchus contortus*) induced by parasite gut antigens. Infect. Immun..

[72] Andrews S., Rolph T., Munn E., Taylort M. (1997). Duration of protective immunity against ovine haemonchosis following vaccination with the nematode gut membrane antigen H11. Res. Vet. Sci..

[73] Bu Y., Jia C., Tian X., Aimulajiang K., Memon M.A., Yan R., Song X., Xu L., Li X. (2020). Immunization of goats with recombinant protein 14-3-3 isoform 2(rHcftt-2) induced moderate protection against *Haemonchus contortus* challenge. Pathogens.

[74] Cox G.N., Pratt D., Hageman R., Boisvenue R.J. (1990). Molecular cloning and primary sequence of a cysteine protease expressed by *Haemonchus contortus* adult worms. Mol. Biochem. Parasitol..

[75] Anbu K.A., Joshi P. (2007). Identification of a 55 kDa *Haemonchus contortus* excretory/secretory glycoprotein as a neutrophil inhibitory factor. Parasite Immunol..

[76] Reinhardt S., Scott I., Simpson H.V. (2011). Neutrophil and eosinophil chemotactic factors in the excretory/secretory products of sheep abomasal nematode parasites: NCF and ECF in abomasal nematodes. Parasitol. Res..

[77] Merkelbach P., Scott I., Khalaf S., Simpson H.V. (2002). Excretory/secretory products of *Haemonchus contortus* inhibit aminopyrine accumulation by rabbit gastric glands in vitro. Vet. Parasitol..

[78] Wang W., Wang S., Zhang H., Yuan C., Yan R., Song X., Xu L., Li X. (2014). Galectin Hco-gal-m from *Haemonchus contortus* modulates goat monocytes and T cell function in different patterns. Parasites Vectors.

[79] Sun Y., Yan R., Muleke C.I., Zhao G., Xu L., Li X. (2006). Recombinant Galectins of *Haemonchus contortus* parasite induces apoptosis in the peripheral blood lymphocytes of goat. Int. J. Pept. Res. Ther..

[80] Wang W., Yuan C., Wang S., Song X., Xu L., Yan R., Hasson I., Li X. (2014). Transcriptional and proteomic analysis reveal recombinant galectins of *Haemonchus contortus* down-regulated functions of goat PBMC and modulation of several signaling cascades in vitro. J. Proteom..

[81] Li Y., Yuan C., Wang L., Lu M., Wang Y., Wen Y., Yan R., Xu L., Song X., Li X. (2016). Transmembrane protein 147 (TMEM147): Another partner protein of *Haemonchus contortus* galectin on the goat peripheral blood mononuclear cells (PBMC). Parasites Vectors.

[82] Yuan C., Zhang H., Wang W., Li Y., Yan R., Xu L., Song X., Li X. (2015). Transmembrane protein 63A is a partner protein of *Haemonchus contortus* galectin in the regulation of goat peripheral blood mononuclear cells. Parasites Vectors.

[83] Lu M., Tian X., Yang X., Yuan C., Ehsan M., Liu X., Yan R., Xu L., Song X., Li X. (2017). The N- and C-terminal carbohydrate recognition domains of *Haemonchus contortus* galectin bind to distinct receptors of goat PBMC and contribute differently to its immunomodulatory functions in host-parasite interactions. Parasites Vectors.

[84] Gadahi J.A., Yongqian B., Ehsan M., Zhang Z.C., Wang S., Yan R.F., Song X.K., Xu L.X., Li X. (2016). *Haemonchus contortus* excretory and secretory proteins (HcESPs) suppress functions of goat PBMCs in vitro. Oncotarget.

[85] Gadahi J.A., Ehsan M., Wang S., Zhang Z., Wang Y., Yan R., Song X., Xu L., Li X. (2016). Recombinant protein of *Haemonchus contortus* 14-3-3 isoform 2 (rHcftt-2) decreased the production of IL-4 and suppressed the proliferation of goat PBMCs in vitro. Exp. Parasitol..

[86] Gadahi J.A., Li B., Ehsan M., Wang S., Zhang Z., Wang Y., Hasan M.W., Yan R., Song X., Xu L. (2016). Recombinant *Haemonchus contortus* 24 kDa excretory/secretory protein (rHcES-24) modulate the immune functions of goat PBMCs in vitro. Oncotarget.

[87] Ehsan M., Gadahi J.A., Hasan M.W., Haseeb M., Ali H., Yan R., Xu L., Song X., Zhu X.-Q., Li X. (2020). Characterization of *Haemonchus contortus* excretory/secretory antigen (ES-15) and its modulatory functions on goat immune cells in vitro. Pathogens.

[88] Ehsan M., Gao W., Gadahi J.A., Lu M., Liu X., Wang Y., Yan R., Xu L., Song X., Li X. (2017). Arginine kinase from *Haemonchus contortus* decreased the proliferation and increased the apoptosis of goat PBMCs in vitro. Parasites Vectors.

[89] Ehsan M., Wang W., Gadahi J.A., Hasan M.W., Lu M., Wang Y., Liu X., Haseeb M., Yan R., Xu L. (2018). The serine/threonine-protein phosphatase 1 from *Haemonchus contortus* is actively involved in suppressive regulatory roles on immune functions of goat peripheral blood mononuclear cells. Front. Immunol..

[90] Wen Y., Wang Y., Wang W., Lu M., Ehsan M., Tian X., Yan R., Song X., Xu L., Li X. (2017). Recombinant Miro domain-containing protein of *Haemonchus contortus* (rMiro-1) activates goat peripheral blood mononuclear cells in vitro. Vet. Parasitol..

[91] Gadahi J.A., Ehsan M., Wang S., Zhang Z., Yan R., Song X., Xu L., Li X. (2017). Recombinant protein of *Haemonchus contortus* small GTPase ADP-ribosylation factor 1 (HcARF1) modulate the cell mediated immune response in vitro. Oncotarget.

[92] Ehsan M., Gadahi J.A., Lu M., Yan R., Xu L., Song X., Zhu X.-Q., Du A., Hu M., Li X. (2020). Recombinant elongation factor 1 alpha of *Haemonchus contortus* affects the functions of goat PBMCs. Parasite Immunol..

[93] Ehsan M., Haseeb M., Hu R.-S., Ali H., Memon M.A., Yan R., Xu L., Song X., Zhu X.-Q., Li X. (2020). Tropomyosin: An excretory/secretory protein from *Haemonchus contortus* mediates the immuno-suppressive potential of goat peripheral blood mononuclear cells in vitro. Vaccines.

[94] Ehsan M., Gadahi J.A., Liu T., Lu M., Wang Y., Hasan M.W., Haseeb M., Yan R., Xu L., Song X. (2020). Identification of a novel methyltransferase-type 12 protein from *Haemonchus contortus* and its effects on functions of goat PBMCs. Parasites Vectors.

[95] Wang Y., Wu L., Liu X., Wang S., Ehsan M., Yan R., Song X., Xu L., Li X. (2017). Characterization of a secreted cystatin of the parasitic nematode *Haemonchus contortus* and its immune-modulatory effect on goat monocytes. Parasites Vectors.

[96] Li B., Gadahi J.A., Gao W., Zhang Z., Ehsan M., Xu L., Song X., Li X., Yan R. (2017). Characterization of a novel aspartyl protease inhibitor from *Haemonchus contortus*. Parasites Vectors.

[97] Charlesworth B. (2009). Effective population size and patterns of molecular evolution and variation. Nat. Rev. Genet..

[98] Gilleard J.S., Redman E. (2016). Genetic diversity and population structure of *Haemonchus contortus*. Adv. Parasitol..

[99] Redman E., Packard E., Grillo V., Smith J., Jackson F., Gilleard J.S. (2008). Microsatellite analysis reveals marked genetic differentiation between *Haemonchus contortus* laboratory isolates and provides a rapid system of genetic fingerprinting. Int. J. Parasitol..

[100] Hunt P., Knox M., Le Jambre L., McNally J., Anderson L. (2008). Genetic and phenotypic differences between isolates of *Haemonchus contortus* in Australia. Int. J. Parasitol..

[101] Poeschel G.P., Todd A.C. (1972). Disease-producing capacity of *Haemonchus contortus* isolates in sheep. Am. J. Vet. Res..

[102] Maizels R.M., Kurniawan A. (2002). Variation and polymorphism in helminth parasites. Parasitology.

[103] Willadsen P. (2008). Antigen cocktails: Valid hypothesis or unsubstantiated hope?. Trends Parasitol..

[104] Lightowlers M.W., Colebrook A., Gauci C.G., Gauci S., Kyngdon C., Monkhouse J., Rodriquez C.V., Read A., Rolfe R., Sato C. (2003). Vaccination against cestode parasites: Anti-helminth vaccines that work and why. Vet. Parasitol..

[105] Barnes E., Dobson R., Barger I. (1995). Worm control and anthelmintic resistance: Adventures with a model. Parasitol. Today.

[106] Ekroth A.K.E., Rafaluk-Mohr C., King K.C. (2019). Host genetic diversity limits parasite success beyond agricultural systems: A meta-analysis. Proc. R. Soc. B Biol. Sci..

[107] Whiteman N.K., Matson K.D., Bollmer J.L., Parker P.G. (2005). Disease ecology in the Galápagos Hawk (*Buteo galapagoensis*): Host genetic diversity, parasite load and natural antibodies. Proc. R. Soc. Lond. B Boil. Sci..

[108] Hoffmann A.A., Sgro C.M. (2011). Climate change and evolutionary adaptation. Nature.

[109] Chauhan N., Tiwari S., Iype T., Jain U. (2017). An overview of adjuvants utilized in prophylactic vaccine formulation as immunomodulators. Expert Rev. Vaccines.

[110] Reed S.G., Orr M.T., Fox C.B. (2013). Key roles of adjuvants in modern vaccines. Nat. Med..

[111] Stutzer C., Richards S.A., Ferreira M., Baron S., Maritz-Olivier C. (2018). Metazoan parasite vaccines: Present status and future prospects. Front. Microbiol..

[112] Cauwelaert N.D., Desbien A.L., Hudson T.E., Pine S.O., Reed S.G., Coler R.N., Orr M.T. (2016). The TLR4 agonist vaccine adjuvant, GLA-SE, requires canonical and atypical mechanisms of action for TH1 induction. PLoS ONE.

[113] Petermann J., Bonnefond R., Mermoud I., Rantoen D., Meynard L., Munro C., Hüe T., Lua L.H.L. (2017). Evaluation of three adjuvants with respect to both adverse effects and the efficacy of antibody production to the Bm86 protein. Exp. Appl. Acarol..

[114] Himly M., Mills-Goodlet R., Geppert M., Duschl A. (2017). Nanomaterials in the context of type 2 immune responses—Fears and potentials. Front. Immunol..

[115] Fawzi E.M., González-Sánchez M.E., Corral M.J., Cuquerella M., Alunda J.M. (2014). Vaccination of lambs against *Haemonchus contortus* infection with a somatic protein (Hc23) from adult helminths. Int. J. Parasitol..

[116] Fawzi E.M., González-Sánchez M.E., Corral M.J., Alunda J.M., Cuquerella M. (2015). Vaccination of lambs with the recombinant protein rHc23 elicits significant protection against *Haemonchus contortus* challenge. Vet. Parasitol..

[117] González-Sánchez M.E., Cuquerella M., Alunda J.M. (2018). Vaccination of lambs against *Haemonchus contortus* with the recombinant rHc23. Effect of adjuvant and antigen dose. PLoS ONE.

[118] Sheerin D., Openshaw P.J.M., Pollard A.J. (2017). Issues in vaccinology: Present challenges and future directions. Eur. J. Immunol..

